# Mortality among male smokers and smokeless tobacco users in the USA

**DOI:** 10.1186/s12954-019-0321-7

**Published:** 2019-08-20

**Authors:** Brad Rodu, Nantaporn Plurphanswat

**Affiliations:** 1 James Graham Brown Cancer Center, University of Louisville, 505 South Hancock Street, Louisville, KY 40202 USA; 2 Department of Medicine, School of Medicine, University of Louisville, Louisville, USA

**Keywords:** Smoking, Smokeless tobacco, Mortality, National Health Interview Survey

## Abstract

**Background:**

One published study simultaneously reported the mortality associated with cigarette smoking and smokeless tobacco (ST) use in the USA. In this study, we focus only on men ages 40–79 years old and extend the follow-up by 4 years.

**Methods:**

We used selected years (1987–2010) of National Health Interview Survey (NHIS) Linked Mortality Files to classify 46,104 men age 40–79 years with respect to 7 categories of smoking and/or ST use. We used Cox proportional hazards models adjusted for age, race/ethnicity, marital status, education, income, health status, body mass index, and region to estimate hazard ratios (HRs; 95% confidence intervals, CI) for mortality from all causes, heart diseases, malignant neoplasms, and two mutually exclusive categories: smoking-related and other diseases.

**Results:**

There were 15,540 deaths from all causes, including 3476 never tobacco users, 4782 exclusive smokers, and 210 exclusive ST users. The latter had significant excess mortality from all causes (HR = 1.25, CI = 1.08–1.46), but not from heart diseases (HR = 1.16, CI = 0.85–1.59), malignant neoplasms (HR = 1.17, CI = 0.83–1.67), and all smoking-related diseases (HR = 1.19, CI = 0.97–1.46). However, they had higher mortality for all other causes (1.39, CI = 1.10–1.74), which was largely seen in age 40–59 years (HR = 1.68, CI = 1.11–2.54). Current smokers, with or without ST use, also had significantly elevated HRs for other causes (1.70 and 1.57, respectively), in addition to significant increases in mortality from heart diseases (1.98 and 2.00), malignant neoplasms (2.60 and 2.84), and all smoking-related diseases (2.32 and 2.47).

**Conclusions:**

This is the first simultaneous mortality follow-up study of older American male smokers and ST users. ST users did not have excess mortality from any smoking-related diseases, but younger users had an elevation in deaths from other causes.

**Electronic supplementary material:**

The online version of this article (10.1186/s12954-019-0321-7) contains supplementary material, which is available to authorized users.

## Background

While cigarette smoking is known to be the leading cause of premature deaths in the USA, much less is known about mortality among smokeless tobacco (ST) users. This deficit of population-level studies is due to the low prevalence of ST use and infrequent collection of ST information in national surveys. The prevalence of ST use among American adult men and women is 4.4% and 0.2%, respectively [1], and National Health Interview Surveys (NHIS), the primary instrument for US smoking rates, collected information about ST use in only nine of the last 30 years.

Previous mortality follow-up studies reported mixed results among ST users. Male exclusive ST users in the first and second Cancer Prevention Studies had significant elevations of 17–18% in all-cause mortality, compared to never tobacco users [2]. In comparison, ST users in the National Longitudinal Mortality Study (NLMS) from 1985 to 2011 had no increased mortality from all causes [3]. An analysis of the National Health and Nutrition Examination Survey I Epidemiologic Follow-up Study through 1992 found no significant associations between current ST use and deaths from all causes [4], and none was reported recently in NLMS and NHIS follow-up analyses through 2011 [5].

Fisher et al. [5] are the first to simultaneously report mortality of ST users and smokers. They found excess mortality among smokers for all causes, diseases of the heart, cerebrovascular disease, malignant neoplasms, chronic lower respiratory diseases, influenza and pneumonia, or diseases of the digestive system; these results were consistent with many previous studies. In contrast, they reported no excess mortality among exclusive ST users for any of these causes. However, Fisher et al. included survey participants 18–39 years, in whom death from any cause is a rare event, regardless of tobacco use. They also included women, among whom ST use is exquisitely rare.

We extended the analysis by Fisher et al. using NHIS Linked Mortality Files from 1987 to 2015, which adds four more years of the follow-up, and restricting the study population to men 40–79 years of age.

## Methods

### Data

We used pooled files from the Integrated Public Use Microdata Series (IPUMS) for NHIS surveys with information on ST use and smoking (1987, 1991, 1992, 1994, 1998, 2000, 2005, and 2010) and linked to the National Death Index [6] to obtain follow-up from year of interview to year of death or December 31, 2015, the censor date for participants who did not die. The NDI is the centralized archive of national death record information obtained from death certificates in 50 states and the District of Columbia. Linkages to NDI are matched via respondents’ social security numbers, first/last names, and birth dates. About 95% of NHIS respondents were eligible for the mortality follow-up. Deaths from all causes and nine leading causes were provided for decedents from NHIS survey years 1986 through 2014. For underlying causes of death, from the 10th revision of International Statistical Classification of Diseases and Related Health Problems (ICD-10) [7], data was provided for decedents from NHIS survey years 1986 through 2004, with follow-up through December 31, 2006.

### Measures

#### Tobacco status

The primary predictor of mortality outcomes was tobacco status at survey enrollment, combining cigarette smoking and use of ST (chewing tobacco and/or snuff). Never smokers had never smoked 100 cigarettes in their lifetime. Current smokers had smoked 100 cigarettes and currently smoked every day or some days. Former smokers had smoked 100 cigarettes but did not currently smoke. Never users of ST had never used chewing tobacco and/or snuff 20 times in their lifetime; current users had used ST 20 times and currently used it every day or some days; former users had used ST 20 times and did not currently use it.

We constructed 7 tobacco status categories: 1—never tobacco users (never smoke, never ST use), 2—never smokers and current ST users (hereafter exclusive current ST users); 3—never smokers and former ST users, 4—current smokers and never/former ST users (hereafter exclusive current smokers), 5—current smokers and current ST users (hereafter dual users), 6—former smokers and never/former ST users, and 7—former smokers and current ST users. Tobacco status 4 and 6 combined never and former ST users to eliminate uninformative combinations. In a recent study, former and never ST users had no statistically significant differences in mortality [3].

Respondents who smoked cigars and/or pipes at survey enrollment were not excluded from our analyses. However, to investigate whether cigar/pipe smoking had any impact on ST results, we created three cigar/pipe categories. Never cigar/pipe smokers had never smoked cigars and/or pipes 50 times in their lifetime; current cigar/pipe smokers had smoked them 50 times and currently smoked them every day or some days; former cigar/pipe smokers had smoked them 50 times and did not currently smoke them. The supplemental analysis including/excluding cigar/pipe smokers involved fewer participants because cigar/pipe questions were not asked in the 1994 survey.

#### Individual characteristics

Demographic and socioeconomic confounders included age, race/ethnicity (non-Hispanic white, non-Hispanic black, other), marital status (never married, married, divorced/separated, widowed), educational attainment (less than high school, high school, some college, college, and higher), family income ($0–$34,999, $35,000–$74,999, ≥ $75,000), self-reported health status (excellent, very good, good, fair, poor), and body mass index (BMI) categories (normal weight, 18.5 ≤ BMI < 25; underweight, BMI < 18.5; overweight, 25 ≤ BMI < 30; obese, BMI ≥ 30). We also included region of residence (Northeast, South, Midwest, West) as well as survey year to capture any variations due to unobservable characteristics.

#### Mortality outcomes

The main outcome measures were mortality from all causes, heart diseases (ICD-10 codes I00–I09, I11, I13, and I20–I51) malignant neoplasms (C00–C97), chronic lower respiratory diseases (J40–J47), and cerebrovascular disease (I60–I69) [7]. In addition, we established a smoking-related disease category by combining heart diseases, malignant neoplasms, chronic lower respiratory and cerebrovascular diseases with diabetes mellitus (E10–E14) and influenza/pneumonia (J09–J18) [7]. Our smoking-related diseases are similar to but broader than those recognized by the Surgeon General [8], because we did not have specific ICD codes (for example, the Surgeon General’s smoking-related cancers are site-specific, whereas ours includes all cancers). Other causes consisted of accidents (V01–X59, Y85–Y86), Alzheimer’s disease (G30), nephritis, nephrotic syndrome and nephrosis (N00–N07, N17–N19, N25–N27), and all other residual causes [7]. Smoking-related diseases and other causes were mutually exclusive and exhaustive.

We examined the association between tobacco status and underlying causes of death from cancer of the trachea, bronchus, and lung (C33–C34); smoking-related cancers such as lip, oral cavity and pharynx (C00–C14), esophagus (C15), larynx (C32), trachea, bronchus and lung, bladder (C67), and leukemia (C91–C95); and digestive system cancers such as cancer of esophagus, pancreas, stomach, colon, rectum, anus (C18–C21), and liver and bile ducts (C22) [7]. Underlying causes of death were not available for 2005 and 2010 survey participants.

#### Study population

The total number of participants for all NHIS survey years was 875,510. Our analyses were restricted a priori to men age 40–79 years (*n* = 57,182). ST is rarely used by women [1], and at younger ages tobacco use may be less stable and deaths are rare [9]. About 97% of these men (*n* = 55,483) were eligible for mortality linkage. Men who died the same year as their survey enrollment accrued no person-years, so they were not eligible for analysis. We limited the sample to men with complete information on smoking and ST use (*n* = 46,538) and we excluded those who had missing race/ethnicity, education, marital status, and self-reported health status. For family income and BMI categories, we included an indicator for missing data in regression models. We applied sample weights adjusted for NDI linkage eligibility in all models.

The final sample size included 46,104 men with 510,684 person-years and 15,845 deaths.

#### Statistical analysis

Cox proportional hazards models were used to examine the associations between tobacco status and mortality outcomes, reported as hazard ratios (HRs; with 95% confidence intervals, CI) with never tobacco users as the referent group. Follow-up was in years between survey enrollment and death or survival until December 31, 2015. Follow-up ranged from 1 to 28 years (mean = 11.1 years; median = 11 years, standard deviation = 6.9 years). For most outcomes, we present results separately for younger (age 40–59 years), older (age 60–79 years), and pooled age groups. For each tobacco use and disease category, we calculated crude death rates per 1000 person-years (the sum of years contributed by all participants in the tobacco use category):

$$ \mathrm{Crude}\ \mathrm{death}\ \mathrm{rate}=\frac{\mathrm{Number}\ \mathrm{of}\ \mathrm{death}\mathrm{s}}{\mathrm{Person}-\mathrm{years}}\times \mathrm{1,000} $$

We then estimated HRs adjusted for age only, then for age, race/ethnicity, marital status, education, income, region, and survey year. The final model added BMI categories and self-reported health status. The rationale for the final model is that BMI and health status may be affected by tobacco status, but these covariates also adjust for other differences that may be correlated with tobacco use and with mortality (i.e., relevant confounders). The addition of BMI and health status did not significantly change the associations between tobacco status and mortality, but they were significantly associated with mortality. Thus, we present the results from the final model as our main results, with results for all three models in Additional file 2.

## Results

### Descriptive statistics

The majority of men (66%) were alive at the end of the follow-up (December 31, 2015). Characteristics of the study participants at survey enrollment, according to tobacco status, are listed in Table 1. Tobacco users were different from never users, most notably in the following ways. The oldest group was former smokers (mean 59 years), who were 5 years older than never users. ST users were more likely to be non-Hispanic white and, along with current and former smokers, have less education and lower family income. Current smokers were less likely than never users to be overweight or obese, while exclusive current ST users were more likely to be obese. All tobacco users were more likely to report fair or poor health. ST users were more likely to live in the South or Midwest.

**Table 1:** Characteristics (unweighted %) of men age 40–79 years at enrollment in NHIS 1987, 1991, 1992, 1994, 1998, 2000, 2005, and 2010^a^

	Never tobacco users	Never smokers		Current smokers		Former smokers		All
		Current ST users	Former ST users	Never + former ST users	Current ST users	Never + former ST users	Current ST users	
Age in year [standard deviation]	54.3 [11.0]	54.0 [11.8]	54.4 [12.0]	53.0 [9.7]	52.5 [10.4]	59.3 [11.0]	58.8 [11.2]	55.9 [11.1]
Race/ethnicity								
Non-Hispanic White	69.7	84.8	82.1	71.7	84.4	8	91.8	74.8
Non-Hispanic Black	12.3	11.6	11.6	16.3	11.8	9.1	5.6	12.0
Other race	18.0	3.6	6.3	12.0	3.8	10.9	2.6	13.2
Marital status								
Never married	13.0	9.6	7.4	12.5	7.0	6.8	6.4	10.3
Married	66.7	65.1	67.9	53.1	56.5	69.8	69.5	64.4
Divorced/separated	16.1	19.7	17.5	28.9	31.2	16.3	17.6	19.6
Widowed	4.2	5.7	7.2	5.4	5.3	7.2	6.5	5.7
Educational attainment								
Less than high school	14.9	33.0	18.3	25.6	39.7	21.0	38.7	20.7
High school	26.7	32.2	29.1	37.0	30.4	31.4	34.4	31.2
Some college	20.8	18.5	23.4	22.9	20.6	22.4	16.4	21.8
College and higher	37.7	16.3	29.3	14.4	9.3	25.2	10.5	26.2
Family income								
$0–24,999	26.3	34.8	25.9	37.9	41.7	31.0	39.4	31.4
$25,000–34,999	9.1	10.4	6.8	11.0	11.8	11.1	10.7	10.3
$35,000–$54,999	22.4	15.8	17.5	21.6	19.6	25.2	20.3	23.0
$55,000–74,999	5.8	6.7	6.7	4.4	3.8	4.9	3.9	5.1
$75,000+	12.1	6.8	14.8	5.7	4.3	8.3	4.4	8.9
Missing income	7.2	10.3	6.5	8.1	7.5	8.3	9.1	7.9
Self-reported health								
Excellent	32.8	23.5	25.3	19.9	18.6	25.3	16.3	26.2
Very good	30.8	28.8	33.1	27.0	19.6	28.1	26.5	28.7
Good	25.0	28.1	27.8	30.1	33.9	28.1	28.4	27.6
Fair	8.4	13.4	9.9	15.9	17.8	13.2	16.7	12.3
Poor	2.9	6.3	4.0	7.2	10.1	5.3	12.1	5.1
BMI categories								
Normal weight	28.3	21.7	18.8	38.0	40.5	26.0	23.8	29.7
Underweight	0.3	0.2	0.0	1.2	1.3	0.3	0.8	0.5
Overweight	45.8	47.1	50.6	40.8	37.2	48.3	46.3	45.4
Obese	21.3	26.9	26.4	16.3	16.3	22.6	26.1	20.7
Missing BMI	4.4	4.1	4.2	3.7	4.8	2.9	3.0	3.7
Region								
Northeast	20.7	7.0	12.4	18.2	6.0	20.8	8.8	19.5
South	33.0	55.7	38.6	36.7	57.3	32.7	54.9	34.8
Midwest	23.3	24.1	27.0	25.1	24.1	24.4	25.1	24.2
West	23.1	13.2	22.1	2	12.6	22.1	11.3	21.5
Observations	15,540	584	526	11,551	398	16,663	842	46,104

^a^Excluding those with missing tobacco status, race/ethnicity, educational attainment, marital status, and/or self-reported health status

*ST* smokeless tobacco

Table 2 presents the number of deaths and crude death rates by tobacco use for all causes and for four of the leading causes that are related to smoking (diseases of the heart, malignant neoplasms, chronic lower respiratory diseases, and cerebrovascular diseases). In addition, it contains deaths and rates for all smoking-related diseases combined and for all other causes, which are exhaustive and mutually exclusive.

**Table 2:** Crude death rate (per 1000 person-years) and number of deaths from all causes and selected leading causes of death among men ages 40–79 years in NHIS 1987, 1991, 1992, 1994, 1998, 2000, 2005, and 2010^a^

	Never tobacco users	Never smokers		Current smokers		Former smokers		All
		Current ST users	Former ST users	Never + former ST users	Current ST users	Never + former ST users	Current ST users	
Number of participants	15,540	584	526	11,551	398	16,663	842	46,104
All causes								
Rate	20.9	32.2	28.4	37.2	39.7	35.0	40.5	31.0
Number	3476	210	139	4782	181	6670	387	15,845
Heart diseases								
Rate	4.6	7.2	9.2	7.6	9.0	8.1	10.3	6.9
Number	760	47	45	983	41	1540	98	3514
Malignant neoplasms								
Rate	5.1	6.4	5.9	12.5	12.5	9.4	11.2	8.8
Number	851	42	29	1613	57	1788	107	4487
Chronic lower respiratory diseases								
Rate	0.4	0.5	0.2	3.2	3.3	2.0	2.5	1.8
Number	64	3	1	408	15	384	24	899
Cerebrovascular diseases								
Rate	1.1	2.2	1.8	1.4	1.1	1.7	2.6	1.4
Number	176	14	9	177	5	325	25	731
Smoking-related diseases^b^								
Rate	12.3	18.3	19.0	26.3	28.1	23.2	28.6	20.5
Number	2048	119	93	3382	128	4415	273	10,458
Other causes, excluding smoking-related diseases^c^								
Rate	8.5	14.0	9.4	10.7	11.6	11.8	11.9	10.5
Number	1414	91	46	1382	53	2243	114	5343

^a^Participants with missing tobacco status, race/ethnicity, educational attainment, marital status or self-reported health status are excluded

^b^Smoking-related diseases: diseases of heart, malignant neoplasms, chronic lower respiratory diseases, cerebrovascular diseases, diabetes mellitus, and influenza and pneumonia

^c^Other causes included accidents, Alzheimer’s disease, nephritis, nephrotic syndrome and nephrosis, and all other causes

*ST* smokeless tobacco. The number of deaths from individual diseases do not add up to the number of deaths from all causes due to unknown cause of death among 43 men (19 age 40–59 years and 24 age 60–79 years)

Additional file 2: Table S1 contains the number of deaths and crude death rates for individual smoking-related and digestive tract cancers among men by tobacco status.

### Tobacco status and mortality

**Fig. 1:**
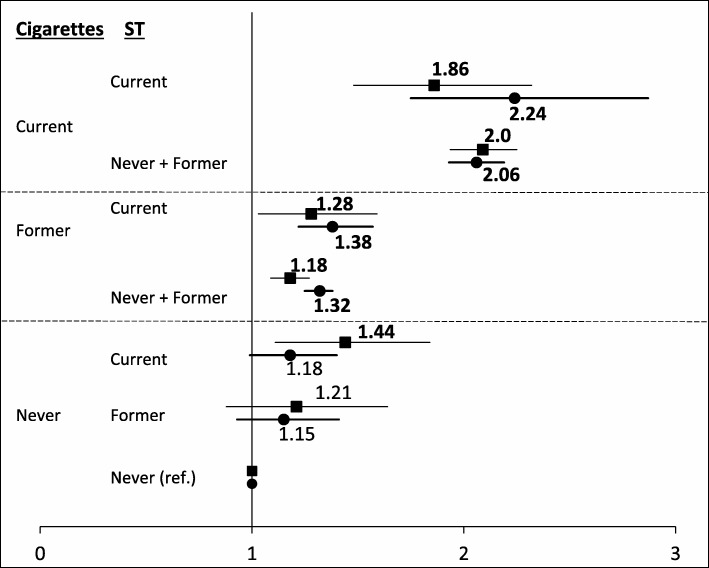
Hazard ratios for all-cause mortality among men age 40–79 years, according to tobacco status. Legend: Squares indicate age 40–59 years. Circles indicate age 60–79 years. Numbers are the point estimates; bold numbers represent statistically significant. Horizontal lines represent 95% confidence interval. ST, smokeless tobacco; ref, referent

Figure 1 presents the associations between tobacco status and mortality from all causes. Significant elevations were seen in both current smokers (HRs = 1.86–2.24) and former smokers (HRs = 1.18–1.38), regardless of age and ST status. Younger exclusive ST users also had significantly increased all-cause mortality (HR = 1.44, CI = 1.12–1.84).

**Fig. 2:**
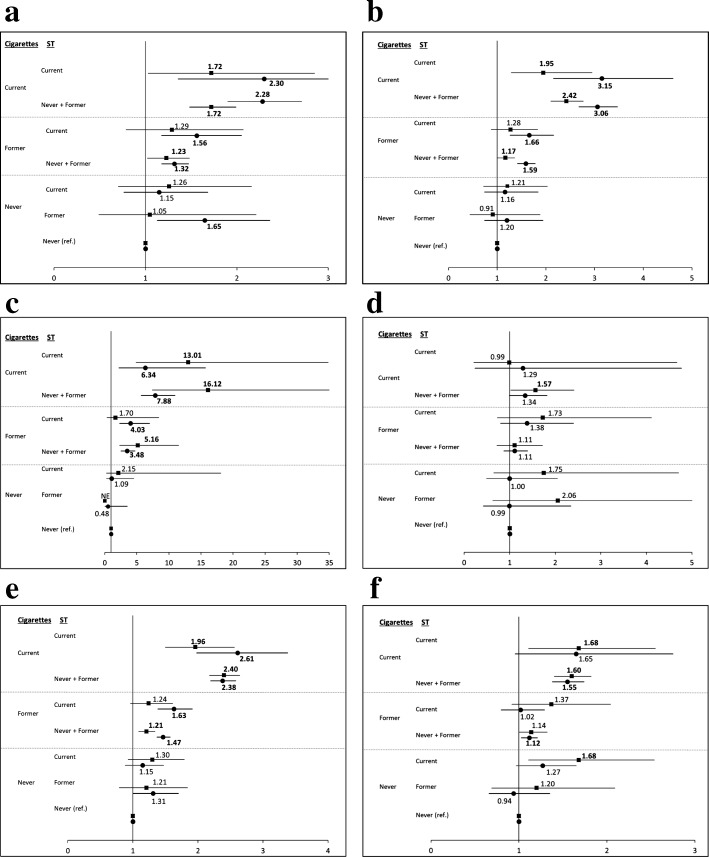
Hazard ratios for specific diseases among men age 40–79 years, according to tobacco status. Legend: **a** heart diseases, **b** malignant neoplasms, **c** chronic lower respiratory diseases, **d** cerebrovascular diseases, **e** smoking-related diseases, and **f** other causes. Squares indicate age 40–59 years. Circles indicate age 60–79 years. Numbers are the point estimates, bold represents statistical significance. Horizontal lines represent 95% confidence interval. Bold numbers represent statistical significance. ST, smokeless tobacco; ref, referent; NE, not estimated; 0 deaths

Figure 2 shows mortality from (a) heart diseases, (b) malignant neoplasms, (c) chronic lower respiratory diseases, (d) cerebrovascular diseases, (e) all smoking-related diseases, and (f) all other causes, according to tobacco use and age group. Exclusive current ST users did not have significant elevations in mortality from any of these diseases, with the exception of all other causes in the younger age group (HR = 1.68, CI = 1.11–2.54).

Current smokers of all ages, with or without ST use, also had elevated HRs for other causes (HRs = 1.55–1.68), in addition to significant increases in mortality from heart diseases (HRs = 1.72–2.30), malignant neoplasms (HRs = 1.95–3.15), chronic lower respiratory diseases (HR = 6.3–16.1), and all smoking-related diseases (HRs = 1.96–2.61). Mortality from these diseases was also higher in former smokers, although point estimates were lower than those in smokers and not always statistically significant.

Additional file 2: Table S2 demonstrates the effect of adjustments in three models for the diseases in Fig. 2: (1) age; (2) model 1 plus race/ethnicity, educational attainment, marital status, family income, region, and survey years; and (3) model 2 plus BMI categories and self-reported health status. Adjustments generally resulted in modest reduction of HR point estimates.

Overall, other covariates among men ages 40–79 had expected associations (results not shown). Compared to never-married men, those who were married had significantly lower all-cause mortality (HR = 0.76, CI = 0.71–0.81). Men with a college diploma or higher had lower mortality than those with less than high school, and all income groups ≥ $35,000 had lower HRs than the referent group ($0–$34,999). Compared with men reporting excellent health, those with progressively poorer self-assessments had increasing HRs in all age groups. Men who were overweight had an advantage in all-cause mortality (HR = 0.94, CI = 0.90–0.97), while those who were obese had an excess of deaths (HR = 1.07, CI = 1.02–1.12).

Additional file 1: Figure S1 provides the associations of tobacco status with mortality from lung, smoking-related, and digestive system cancers. HRs for these cancers were not significantly elevated in exclusive current or former ST users. Current smokers had 8–12-fold increases in mortality from lung cancer, while former smokers’ HRs were 4 times larger than never users. Smoking-related cancer deaths were also significantly elevated in current and former smokers (HRs = 4.4 to 6.5 and 2.5–3.0, respectively).

The results for all-cause mortality, including or excluding cigar and pipe use, are compared in Additional file 2: Table S3. Current cigar/pipe use confers higher all-cause mortality in younger men (HR = 1.17, CI = 1.05–1.30) but not in older men or in all ages combined. Models excluding or including adjustment for cigar/pipe use produced similar HRs for all tobacco users.

## Discussion

By focusing only on men age 40–79 years at survey enrollment, this study produced a more informative appraisal of the health effects of ST use and smoking, compared with other similar studies. By excluding women and younger survey participants, our analytic sample was restricted to a population with long and stable exposure to ST and cigarettes, and all subjects were at risk during follow-up for relevant disease outcomes. In addition, we obtained recently released data extending follow-up from 2011 through 2015 (as long as 28 years).

Our primary finding is that exclusive current ST users had significantly elevated all-cause mortality (HR = 1.25, CI = 1.08–1.46), which was largely driven by elevated mortality from other causes in the younger age group (HR = 1.68, CI = 1.11–2.54). Although we did not find statistically significant increases in mortality from heart diseases, malignant neoplasms, and all smoking-related diseases, the HRs for these causes were elevated by about 20%.

Henley et al. also reported excess mortality from other causes among ST users in the first (HR = 1.17, CI = 1.06–1.30) and second (HR = 1.11, CI = 0.97–1.25) Cancer Prevention Studies (CPS) in addition to elevated HRs for smoking-related diseases [2]. However, in our study, ST users did not have elevated HRs for the latter, and the increase in other causes was only found in the younger age group. It is possible that younger ST users in this study have unobserved differences in behaviors and/or lifestyle that contribute to deaths from causes unrelated to any form of tobacco use. Confirmation will require additional investigation, including extended follow-up and further analysis of underlying cause of death.

Younger and older smokers in this study also had significantly increased mortality from other causes (HRs = 1.66–2.32), which may be due to diseases that have not been formally linked to smoking but have credible pathophysiologic pathways [8, 9]. Our findings are consistent with a recent study of smokers age 35+ years using restricted NHIS linked mortality files that had underlying cause of death for all decedents [8]. Those authors found that 20% of excess deaths were due to causes other than the 21 formally established by the US Surgeon General as smoking-attributable. Our smoking-related disease category was less comprehensive. Other causes made up about 20% of excess deaths among current and former smokers in our study. Another large pooled analysis showed that male smokers age 55+ years have increased mortality from at least 14 other causes not currently attributable to smoking, comprising 16% of excess mortality [10]. All-cause mortality among exclusive current ST users was in this study (HR = 1.25) was similar in magnitude to Henley et al. (HRs = 1.17, CI = 1.11–1.23 and 1.18, CI = 1.08–1.29, respectively) [2]. Other previous studies produced null results for all-cause mortality [3–5], although the latter two studies included both men and women.

We did not observe any significant association between current ST use and cardiovascular diseases or all cancers, which was similar to Accortt et al. [4] and Fisher et al. [5]. In contrast, Timberlake et al. reported increased deaths for coronary heart diseases [3], and Henley et al. found that current ST users in CPS-I had significant excess deaths from digestive system cancers, cardiovascular diseases, and digestive system diseases, while those in CPS-II had excess mortality from all cancers, lung and other cancers, and cardiovascular diseases [2].

Current ST use was not associated with excess mortality from malignancies attributable to smoking or ST use. The negative findings for digestive system cancers are consistent with several studies of snus users and smokers in Sweden [11–13] and one in the USA [14]. Another Swedish study was the exception, finding that snus users had elevated risk for esophageal cancer [15].

Strengths of the current study include a population-based sample and a large number of participants at enrollment with information about multiple forms of tobacco use, important socioeconomic variables and the 10 leading causes of death. Nevertheless, there are limitations that may affect our findings. First, tobacco use information was collected only at baseline and did not reflect changes that may have occurred over time. Second, the lack of information on amount and duration of consumption among current users and years since quit among former users could alter our findings. Lariscy et al. confirmed recently that current smokers with higher consumption have higher mortality rates and that mortality rates decline in former smokers as years since quit smoking increase [9]. Third, we have limited statistical power and high standard errors due to low prevalence of ST use and low numbers of deaths. As a result, we may not have detected significant associations for some less common causes of death. Fourth, the NHIS did not have information on preexisting conditions in survey years in our study. However, we excluded participants who died the same year as their interview. Finally, adjustment for other risk factors that were not available, including alcohol consumption, diet, physical activity, hypertension, and diabetes, could have potentially modified the results for tobacco users.

The most important limitation was the lack of specific ICD codes for other causes of death, which were the driver for excess mortality among younger ST users. Further investigation of these deaths requires access to restricted mortality data. Even then, the relatively small numbers of deaths from other causes among young ST users (*n* = 31) may preclude a satisfactory explanation.

## Conclusions

This study provides the first analyses of excess mortality among male ST users and smokers at ages when smoking-related deaths occur. ST use is associated with 25% higher all-cause mortality than never users, but there is no convincing evidence that the excess is linked to cardiovascular diseases, malignant neoplasms, or smoking-related diseases. Rather, it is mainly due to excess mortality from other causes, especially in younger ST users.

## Additional files

Additional file 1: **Figure S1.** Hazard ratios for selected cancers among men age 40–79 years according to tobacco status. Legend: (a) trachea, bronchus and lung; (b) smoking-related (includes lip, oral cavity, pharynx, esophagus, pancreas, larynx, trachea, bronchus, lung, bladder and leukemia); and (c) digestive system (includes esophagus, pancreas, stomach, colon, rectum, anus, liver and bile ducts). Squares indicate age 40–59 years. Circles indicate age 60–79 years. Numbers are the point estimates, bold numbers represent statistically significant. Horizontal lines represent 95% confidence interval. ST—smokeless tobacco, ref—referent, NE—not estimated, 0 deaths. (DOCX 96 kb)

Additional file 2: **Table S1.** Crude death rates (per 1000 person-years) and number of deaths from selected cancers among men age 40–79 years in NHIS 1987, 1991, 1992, 1994, 1998, and 2000. **Table S2.** Hazard ratios^a^ of all-cause and cause-specific mortality associated with tobacco users among men age 40–79 years in NHIS 1987, 1991, 1992, 1994, 1998, 2000, 2005, and 2010^b^. **Table S3.** Hazard ratios^a^ of all-cause mortality associated with tobacco users among men age 40–79 years in NHIS 1987, 1991, 1992, 1998, 2000, and 2005^b^. (DOCX 43 kb)

## Data Availability

The datasets are publicly available from IPUMS at 10.18128/D070.V6.3.

## Abbreviations

BMI: Body mass index
CI: Confidence interval
CPS: Cancer Prevention Study
HR: Hazard ratio
ICD: International Classification of Diseases and Related Health Problems
IPUMS: Integrated Public Use Microdata Series
NDI: National Death Index
NHIS: National Health Interview Survey
NLMS: National Longitudinal Mortality Study
ST: Smokeless tobacco

## References

[1] Phillips E, Wang TW, Husten CG (2017). Tobacco product use among adults — United States, 2015. MMWR Morb Mortal Wkly Rep.

[2] Henley SJ, Thun MJ, Connell C, Calle EE (2005). Two large prospective studies of mortality among men who use snuff or chewing tobacco (United States). Cancer Causes Control.

[3] Timberlake DS, Nikitin D, Johnson NJ, Altekruse SF (2017). A longitudinal study of smokeless tobacco use and mortality in the United States. Int J Cancer.

[4] Accortt NA, Waterbor JW, Beall C, Howard G (2002). Chronic disease mortality in a cohort of smokeless tobacco users. Am J Epidemiol.

[5] Fisher M, Tan-Torres SM, Gaworski CL, Black RA, Sarkar MA (2019). Smokeless tobacco mortality risks: an analysis of two contemporary nationally representative longitudinal mortality studies. Harm Red J.

[6] Blewett LA, Drew JAR, Griffin R, King ML, Williams KCW (2018). IPUMS Health Surveys: National Health Interview Survey, version 6.3 [dataset].

[7] Anderson RN, Miniño AM, Hoyert DL, Rosenberg HM (2001). Comparability of cause of death between ICD–9 and ICD–10: preliminary estimates. National Vital Statistics Reports: 46.2.

[8] U.S. Department of Health and Human Services (2014). The health consequences of smoking: 50 years of progress. A report of the Surgeon General.

[9] Lariscy JT, Hummer RA, Rogers RG (2018). Cigarette smoking and all-cause and cause-specific adult mortality in the United States. Demography.

[10] Carter BD, Abnet CC, Feskanich D, Freedman ND, Hartge P, Lewis CE (2015). Smoking and mortality – beyond established causes. NEJM.

[11] Ye YE, Ekström AM, Hansson L-E, Bergström R, Nyrén O (1999). Tobacco, alcohol and the risk of gastric cancer by sub-site and histologic type. Int J Cancer.

[12] Lagergren J, Bergström R, Lindgren A, Nyrén O (2000). The role of tobacco, snuff and alcohol use in the aetiology of cancer of the oesophagus and gastric cardia. Int J Cancer.

[13] Nordenvall C, Nilsson PJ, Ye W, Nyrén O (2010). Smoking, snus use and risk of right- and left-sided colon, rectal and anal cancer: a 37-year follow-up study. Int J Cancer.

[14] Chao A, Thun MJ, Henley SJ, Jacobs EJ, McCullough ML, Calle EE (2002). Cigarette smoking, use of other tobacco products and stomach cancer mortality in US adults: the Cancer Prevention Study II. Int J Cancer.

[15] Zendehdel K, Nyrén O, Luo J, Dickman PW, Boffetta P, Englund A, Ye W (2008). Risk of gastroesophageal cancer among smokers and users of Scandinavian moist snuff. Int J Cancer.

