# Supplementary material for: Mortality among male smokers and smokeless tobacco users in the USA
Source: Harm Reduct J. 2019 Aug 20;16:50. doi: 10.1186/s12954-019-0321-7 (PMC6701144; doi:10.1186/s12954-019-0321-7)
Supplement: Supplementary file 1 — Figure S1. Hazard ratios for selected cancers among men age 40–79 years according to tobacco status. Legend: (a) trachea, bronchus and lung; (b) smoking-related (includes lip, oral cavity, pharynx, esophagus, pancreas, larynx, trachea, bronchus, lung, bladder and leukemia); and (c) digestive system (includes esophagus, pancreas, stomach, colon, rectum, anus, liver and bile ducts). Squares indicate age 40–59 years. Circles indicate age 60–79 years. Numbers are the point estimates, bold numbers represent statistically significant. Horizontal lines represent 95% confidence interval. ST—smokeless tobacco, ref—referent, NE—not estimated, 0 deaths. (DOCX 96 kb) [file 12954_2019_321_MOESM1_ESM.docx]

| **a** |  | **b** |
| --- | --- | --- |
|  |  |  |
| 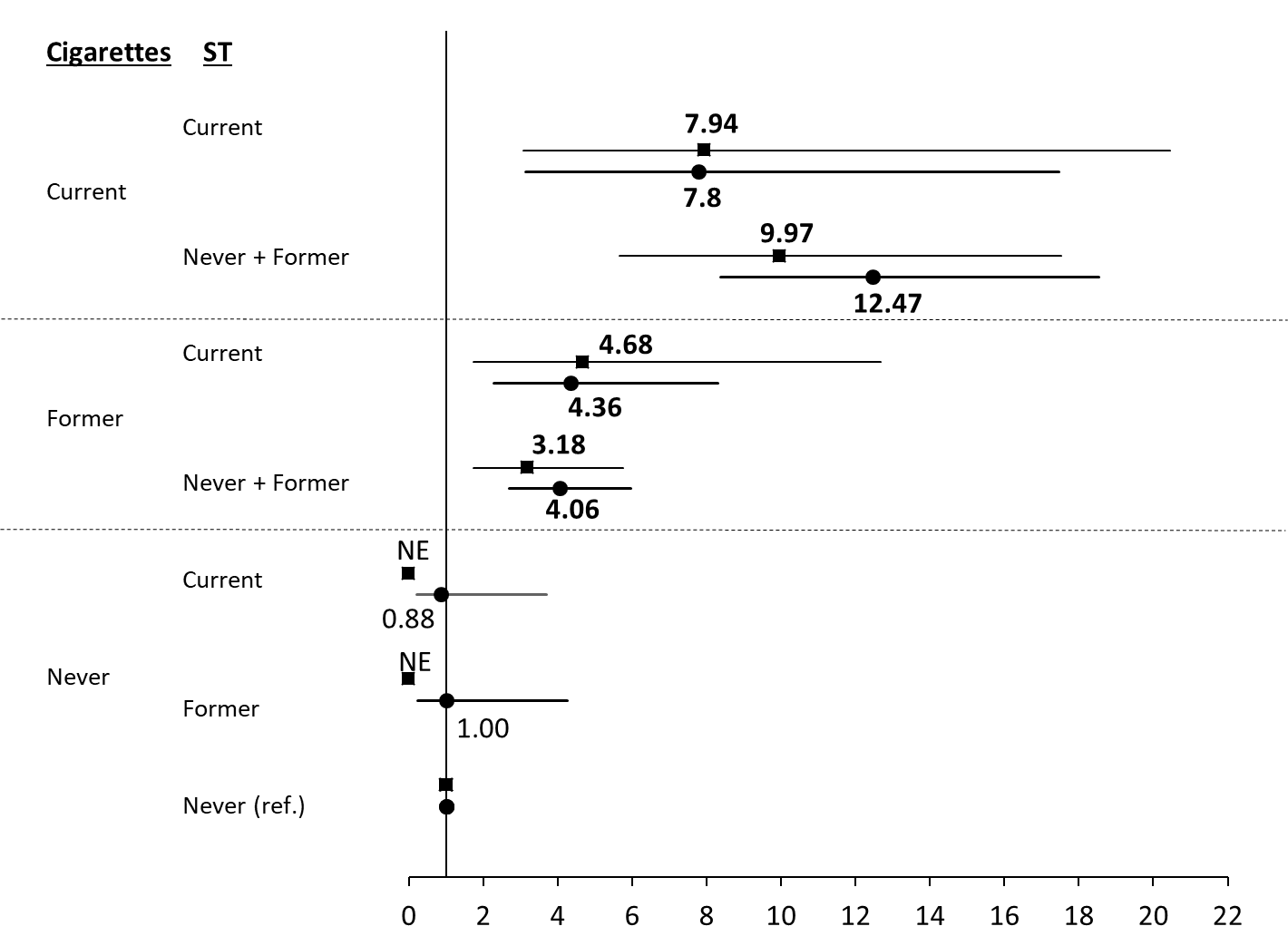 |  | 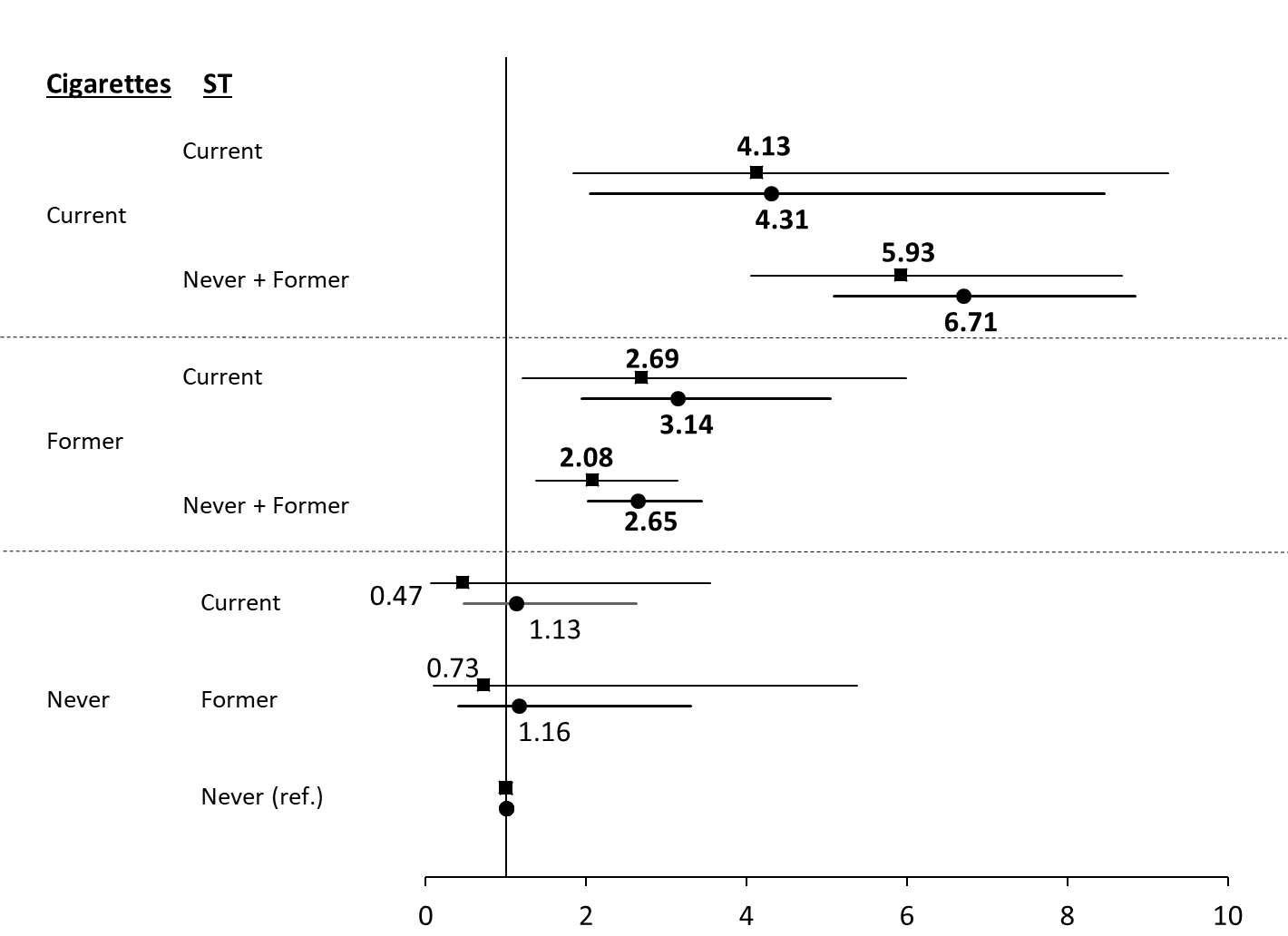 |
|  |  |  |
| **c** |  |  |
|  |  |  |
| 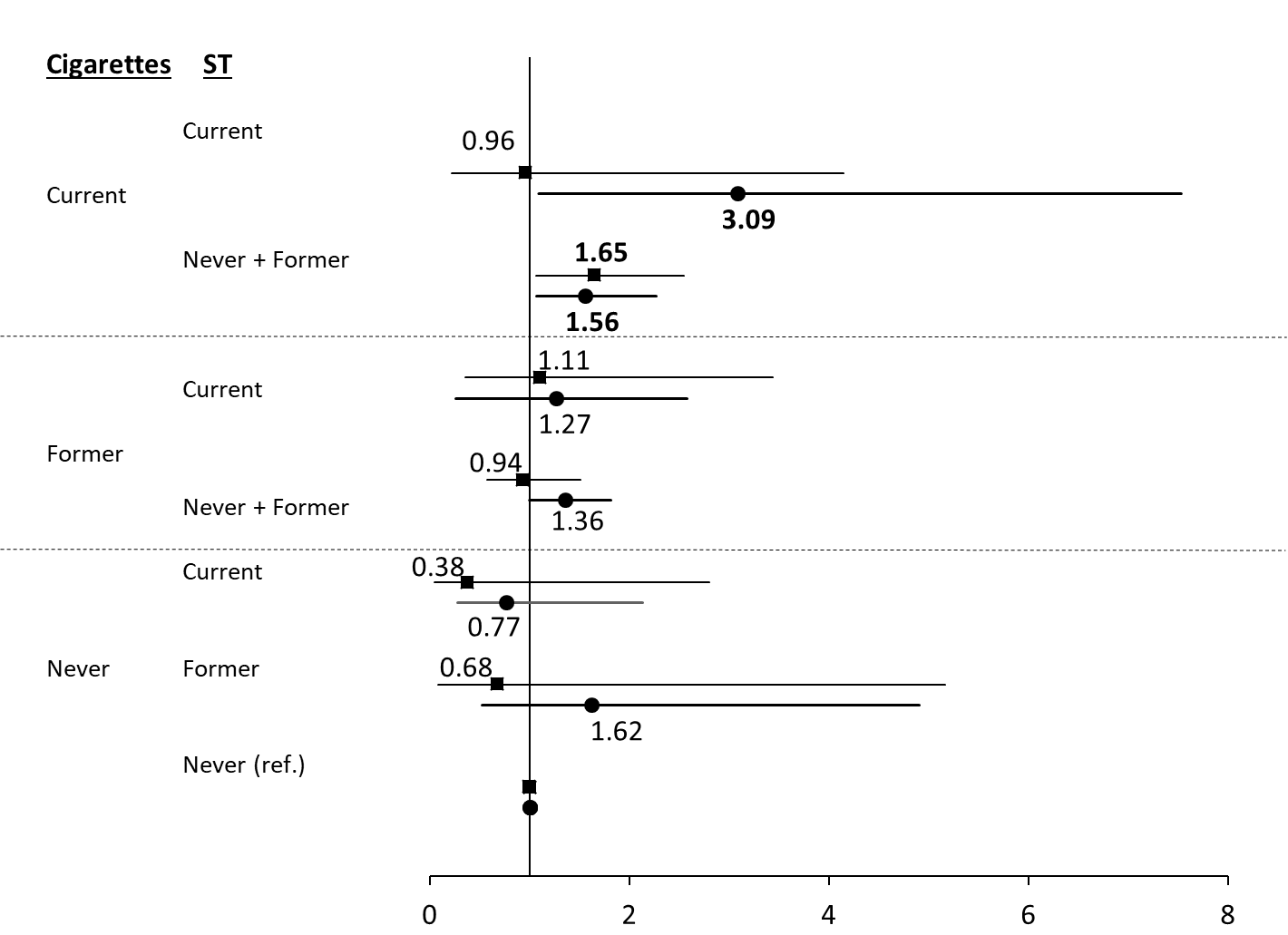 |  |  |
|  |  |  |

All results are weighted. Participants with missing tobacco status, race/ethnicity, educational attainment, marital status or self-reported health status are excluded.

Hazard ratio adjusted for age, race/ethnicity, educational attainment, marital status, family income, BMI categories, self-report health status, region, and survey years.

NE, not estimated; the number of deaths = 0.
