# Supplementary material for: Mortality among male smokers and smokeless tobacco users in the USA
Source: Harm Reduct J. 2019 Aug 20;16:50. doi: 10.1186/s12954-019-0321-7 (PMC6701144; doi:10.1186/s12954-019-0321-7)
Supplement: Supplementary file 2 — Table S1. Crude death rates (per 1000 person-years) and number of deaths from selected cancers among men age 40–79 years in NHIS 1987, 1991, 1992, 1994, 1998, and 2000. Table S2. Hazard ratiosa of all-cause and cause-specific mortality associated with tobacco users among men age 40–79 years in NHIS 1987, 1991, 1992, 1994, 1998, 2000, 2005, and 2010b. Table S3. Hazard ratiosa of all-cause mortality associated with tobacco users among men age 40–79 years in NHIS 1987, 1991, 1992, 1998, 2000, and 2005b. (DOCX 43 kb) [file 12954_2019_321_MOESM2_ESM.docx]

Supplemental Tables

Supplemental Table 1. Crude death rates (per 1,000 person-years) and number of deaths from selected cancers among men age 40-79 years in NHIS 1987, 1991, 1992, 1994, 1998 and 2000

| Cancer | Never smoke | | | Current smoke | | Former smoke | | All |
| --- | --- | --- | --- | --- | --- | --- | --- | --- |
|  | Never ST ^a^ use | Current ST use | Former ST use | Never + former ST use | Current ST use | Never + former ST use | Current ST use |  |
| Lip, oral cavity and pharynx |  |  |  |  |  |  |  |  |
| Rate | 0.01 | 0.17 | 0 | 0.25 | 0 | 0.05 | 0.23 | 0.09 |
| Number | 2 | 1 | 0 | 29 | 0 | 8 | 2 | 42 |
| Esophagus |  |  |  |  |  |  |  |  |
| Rate | 0.09 | 0.17 | 0.25 | 0.22 | 0.24 | 0.21 | 0.23 | 0.18 |
| Number | 13 | 1 | 1 | 26 | 1 | 36 | 2 | 80 |
| Stomach |  |  |  |  |  |  |  |  |
| Rate | 0.05 | 0 | 0 | 0.09 | 0.48 | 0.12 | 0 | 0.09 |
| Number | 7 | 0 | 0 | 10 | 2 | 20 | 0 | 39 |
| Colon, rectum, and anus |  |  |  |  |  |  |  |  |
| Rate | 0.39 | 0.51 | 0.49 | 0.44 | 0.24 | 0.58 | 0.8 | 0.49 |
| Number | 57 | 3 | 2 | 51 | 1 | 101 | 7 | 222 |
| Liver and bile ducts |  |  |  |  |  |  |  |  |
| Rate | 0.03 | 0 | 0.25 | 0.14 | 0.24 | 0.14 | 0.23 | 0.11 |
| Number | 5 | 0 | 1 | 16 | 1 | 25 | 2 | 50 |
| Pancreas |  |  |  |  |  |  |  |  |
| Rate | 0.20 | 0.17 | 0.25 | 0.30 | 0.48 | 0.24 | 0.34 | 0.25 |
| Number | 29 | 1 | 1 | 35 | 2 | 41 | 3 | 112 |
| Larynx |  |  |  |  |  |  |  |  |
| Rate | 0 | 0 | 0 | 0.09 | 0 | 0.05 | 0.11 | 0.04 |
| Number | 0 | 0 | 0 | 10 | 0 | 8 | 1 | 19 |
| Trachea, bronchus and lung |  |  |  |  |  |  |  |  |
| Rate | 0.33 | 0.34 | 0.49 | 3.82 | 3.58 | 1.73 | 2.40 | 1.82 |
| Number | 48 | 2 | 2 | 443 | 15 | 299 | 21 | 830 |
| Bladder |  |  |  |  |  |  |  |  |
| Rate | 0.05 | 0 | 0 | 0.14 | 0 | 0.2 | 0.34 | 0.13 |
| Number | 7 | 0 | 0 | 16 | 0 | 35 | 3 | 61 |
| Leukemia |  |  |  |  |  |  |  |  |
| Rate | 0.10 | 0.34 | 0.25 | 0.15 | 0 | 0.27 | 0.11 | 0.18 |
| Number | 15 | 2 | 1 | 17 | 0 | 46 | 1 | 82 |
| Smoking-related cancers ^b^ |  |  |  |  |  |  |  |  |
| Rate | 0.79 | 1.19 | 1.23 | 4.96 | 4.29 | 2.74 | 3.78 | 2.68 |
| Number | 114 | 7 | 5 | 576 | 18 | 473 | 33 | 1,226 |
| Digestive system cancers ^c^ |  |  |  |  |  |  |  |  |
| Rate | 0.77 | 0.85 | 1.23 | 1.19 | 1.67 | 1.29 | 1.6 | 1.1 |
| Number | 111 | 5 | 5 | 138 | 7 | 223 | 14 | 503 |

^a^ ST, smokeless tobacco

^b^ Smoking-related cancers: lip, oral cavity, pharynx, esophagus, pancreas, larynx, trachea, bronchus, lung, bladder, and leukemia

^c^ Digestive system cancers: esophagus, pancreas, stomach, colon, rectum, anus, and liver and bile ducts.

Supplemental Table 2. Hazard ratios ^a^ of all-cause and cause-specific mortality associated with tobacco users among men age 40-79 years in NHIS 1987, 1991, 1992, 1994, 1998, 2000, 2005, and 2010 ^b^

|  |  | **Age 40-59** | | | **Age 60-79** | | | **Age 40-79** | | |
| --- | --- | --- | --- | --- | --- | --- | --- | --- | --- | --- |
|  |  | **(1)^c^** | **(2)^d^** | **(3)^e^** | **(1)** | **(2)** | **(3)** | **(1)** | **(2)** | **(3)** |
| **All-cause mortality** | |  |  |  |  |  |  |  |  |  |
| Smoking | ST |  |  |  |  |  |  |  |  |  |
| Never | Never | Ref. | Ref. | Ref. | Ref. | Ref. | Ref. | Ref. | Ref. | Ref. |
|  |  |  |  |  |  |  |  |  |  |  |
|  |  |  |  |  |  |  |  |  |  |  |
| Never | Current | 1.59 | 1.47 | 1.44 | 1.40 | 1.25 | 1.18 | 1.47 | 1.32 | 1.25 |
|  |  | [1.24,2.04] | [1.16,1.87] | [1.12,1.84] | [1.19,1.64] | [1.06,1.48] | [0.99,1.40] | [1.27,1.68] | [1.14,1.52] | [1.08,1.46] |
|  |  |  |  |  |  |  |  |  |  |  |
| Never | Former | 1.33 | 1.26 | 1.21 | 1.20 | 1.15 | 1.15 | 1.23 | 1.19 | 1.17 |
|  |  | [0.94,1.89] | [0.91,1.74] | [0.89,1.64] | [0.98,1.47] | [0.94,1.42] | [0.93,1.41] | [1.03,1.48] | [0.99,1.43] | [0.98,1.40] |
|  |  |  |  |  |  |  |  |  |  |  |
| Current | Never + former | 2.62 | 2.28 | 2.09 | 2.31 | 2.17 | 2.06 | 2.48 | 2.26 | 2.11 |
|  |  | [2.43,2.81] | [2.12,2.45] | [1.94,2.25] | [2.17,2.46] | [2.04,2.31] | [1.93,2.19] | [2.37,2.60] | [2.16,2.37] | [2.01,2.22] |
|  |  |  |  |  |  |  |  |  |  |  |
| Current | Current | 2.46 | 2.09 | 1.86 | 2.72 | 2.45 | 2.24 | 2.63 | 2.31 | 2.07 |
|  |  | [1.96,3.10] | [1.68,2.62] | [1.48,2.32] | [2.16,3.42] | [1.97,3.06] | [1.75,2.87] | [2.24,3.08] | [1.98,2.70] | [1.76,2.43] |
|  |  |  |  |  |  |  |  |  |  |  |
| Former | Never + former | 1.15 | 1.21 | 1.18 | 1.37 | 1.35 | 1.32 | 1.31 | 1.31 | 1.28 |
|  |  | [1.06,1.25] | [1.12,1.31] | [1.09,1.27] | [1.30,1.44] | [1.28,1.41] | [1.25,1.38] | [1.26,1.37] | [1.26,1.37] | [1.23,1.34] |
|  |  |  |  |  |  |  |  |  |  |  |
| Former | Current | 1.44 | 1.42 | 1.28 | 1.65 | 1.45 | 1.38 | 1.59 | 1.42 | 1.34 |
|  |  | [1.16,1.79] | [1.14,1.76] | [1.03,1.59] | [1.46,1.87] | [1.28,1.65] | [1.22,1.57] | [1.42,1.77] | [1.27,1.59] | [1.20,1.49] |
|  |  |  |  |  |  |  |  |  |  |  |
| **Heart diseases** | |  |  |  |  |  |  |  |  |  |
| Smoking | ST |  |  |  |  |  |  |  |  |  |
| Never | Never | Ref. | Ref. | Ref. | Ref. | Ref. | Ref. | Ref. | Ref. | Ref. |
|  |  |  |  |  |  |  |  |  |  |  |
|  |  |  |  |  |  |  |  |  |  |  |
| Never | Current | 1.42 | 1.31 | 1.26 | 1.46 | 1.24 | 1.15 | 1.45 | 1.25 | 1.16 |
|  |  | [0.83,2.43] | [0.77,2.24] | [0.73,2.16] | [1.02,2.09] | [0.86,1.79] | [0.78,1.68] | [1.07,1.95] | [0.93,1.70] | [0.85,1.59] |
|  |  |  |  |  |  |  |  |  |  |  |
| Never | Former | 1.11 | 1.11 | 1.05 | 1.92 | 1.73 | 1.65 | 1.69 | 1.60 | 1.51 |
|  |  | [0.53,2.36] | [0.53,2.34] | [0.49,2.21] | [1.34,2.75] | [1.22,2.47] | [1.15,2.36] | [1.23,2.33] | [1.16,2.19] | [1.10,2.08] |
|  |  |  |  |  |  |  |  |  |  |  |
| Current | Never + former | 2.79 | 2.43 | 2.28 | 2.01 | 1.81 | 1.72 | 2.38 | 2.11 | 2.00 |
|  |  | [2.35,3.30] | [2.04,2.88] | [1.91,2.71] | [1.74,2.31] | [1.57,2.08] | [1.49,1.99] | [2.14,2.64] | [1.89,2.34] | [1.79,2.23] |
|  |  |  |  |  |  |  |  |  |  |  |
| Current | Current | 2.16 | 1.86 | 1.72 | 3.11 | 2.56 | 2.30 | 2.60 | 2.19 | 1.98 |
|  |  | [1.31,3.56] | [1.12,3.10] | [1.03,2.85] | [1.94,4.98] | [1.58,4.14] | [1.40,3.76] | [1.85,3.66] | [1.55,3.09] | [1.40,2.80] |
|  |  |  |  |  |  |  |  |  |  |  |
| Former | Never + former | 1.22 | 1.29 | 1.23 | 1.37 | 1.36 | 1.32 | 1.34 | 1.34 | 1.30 |
|  |  | [1.01,1.46] | [1.07,1.54] | [1.02,1.48] | [1.23,1.53] | [1.22,1.52] | [1.18,1.47] | [1.22,1.48] | [1.22,1.48] | [1.18,1.43] |
|  |  |  |  |  |  |  |  |  |  |  |
| Former | Current | 1.47 | 1.47 | 1.29 | 1.94 | 1.69 | 1.56 | 1.83 | 1.63 | 1.50 |
|  |  | [0.92,2.34] | [0.92,2.35] | [0.81,2.07] | [1.48,2.53] | [1.28,2.22] | [1.19,2.05] | [1.45,2.30] | [1.29,2.06] | [1.18,1.89] |
|  |  |  |  |  |  |  |  |  |  |  |
|  |  |  |  |  |  |  |  |  |  |  |
| **Malignant neoplasms** | |  |  |  |  |  |  |  |  |  |
| Smoking | ST |  |  |  |  |  |  |  |  |  |
| Never | Never | Ref. | Ref. | Ref. | Ref. | Ref. | Ref. | Ref. | Ref. | Ref. |
|  |  |  |  |  |  |  |  |  |  |  |
|  |  |  |  |  |  |  |  |  |  |  |
| Never | Current | 1.27 | 1.22 | 1.21 | 1.36 | 1.21 | 1.16 | 1.33 | 1.20 | 1.17 |
|  |  | [0.74,2.18] | [0.72,2.05] | [0.72,2.03] | [0.86,2.14] | [0.77,1.91] | [0.74,1.84] | [0.93,1.88] | [0.85,1.71] | [0.83,1.67] |
|  |  |  |  |  |  |  |  |  |  |  |
| Never | Former | 1.02 | 0.92 | 0.91 | 1.24 | 1.19 | 1.20 | 1.11 | 1.06 | 1.06 |
|  |  | [0.49,2.14] | [0.45,1.89] | [0.44,1.88] | [0.77,1.99] | [0.74,1.93] | [0.74,1.94] | [0.73,1.66] | [0.70,1.59] | [0.70,1.59] |
|  |  |  |  |  |  |  |  |  |  |  |
| Current | Never + former | 2.84 | 2.60 | 2.42 | 3.33 | 3.17 | 3.06 | 3.20 | 3.00 | 2.84 |
|  |  | [2.49,3.24] | [2.27,2.97] | [2.11,2.77] | [2.93,3.78] | [2.79,3.60] | [2.69,3.47] | [2.92,3.51] | [2.73,3.29] | [2.58,3.12] |
|  |  |  |  |  |  |  |  |  |  |  |
| Current | Current | 2.36 | 2.14 | 1.95 | 3.76 | 3.29 | 3.15 | 3.16 | 2.80 | 2.60 |
|  |  | [1.56,3.55] | [1.42,3.23] | [1.29,2.95] | [2.56,5.53] | [2.26,4.80] | [2.16,4.61] | [2.39,4.18] | [2.12,3.70] | [1.96,3.44] |
|  |  |  |  |  |  |  |  |  |  |  |
| Former | Never + former | 1.14 | 1.18 | 1.17 | 1.67 | 1.62 | 1.59 | 1.48 | 1.46 | 1.43 |
|  |  | [0.98,1.32] | [1.02,1.37] | [1.01,1.36] | [1.49,1.86] | [1.45,1.81] | [1.42,1.78] | [1.35,1.61] | [1.33,1.59] | [1.31,1.57] |
|  |  |  |  |  |  |  |  |  |  |  |
| Former | Current | 1.38 | 1.34 | 1.28 | 1.99 | 1.73 | 1.66 | 1.76 | 1.56 | 1.50 |
|  |  | [0.96,1.98] | [0.93,1.93] | [0.89,1.83] | [1.52,2.61] | [1.32,2.25] | [1.28,2.16] | [1.41,2.19] | [1.26,1.94] | [1.21,1.86] |
|  | |  |  |  |  |  |  |  |  |  |
| **Chronic lower respiratory diseases** | |  |  |  |  |  |  |  |  |  |
| Smoking | ST |  |  |  |  |  |  |  |  |  |
| Never | Never | Ref. | Ref. | Ref. | Ref. | Ref. | Ref. | Ref. | Ref. | Ref. |
|  |  |  |  |  |  |  |  |  |  |  |
|  |  |  |  |  |  |  |  |  |  |  |
| Never | Current | 2.99 | 2.14 | 2.15 | 1.43 | 1.11 | 1.09 | 1.66 | 1.24 | 1.23 |
|  |  | [0.36,24.67] | [0.26,17.78] | [0.26,18.11] | [0.34,5.92] | [0.26,4.65] | [0.27,4.50] | [0.51,5.41] | [0.38,4.07] | [0.38,4.01] |
|  |  |  |  |  |  |  |  |  |  |  |
| Never | Former | NE | NE | NE | 0.42 | 0.41 | 0.48 | 0.37 | 0.34 | 0.38 |
|  |  |  |  |  | [0.06,3.05] | [0.06,2.94] | [0.07,3.48] | [0.05,2.66] | [0.05,2.46] | [0.05,2.78] |
|  |  |  |  |  |  |  |  |  |  |  |
| Current | Never + former | 24.65 | 18.97 | 16.12 | 10.84 | 9.65 | 7.88 | 13.58 | 11.55 | 9.49 |
|  |  | [11.32,53.69] | [8.81,40.85] | [7.40,35.13] | [7.91,14.85] | [7.02,13.24] | [5.68,10.92] | [10.21,18.05] | [8.67,15.37] | [7.09,12.72] |
|  |  |  |  |  |  |  |  |  |  |  |
| Current | Current | 24.36 | 14.86 | 13.01 | 9.92 | 8.01 | 6.34 | 13.43 | 9.82 | 7.52 |
|  |  | [8.80,67.47] | [5.44,40.57] | [4.86,34.84] | [4.29,22.92] | [3.42,18.77] | [2.56,15.70] | [7.41,24.33] | [5.38,17.90] | [4.00,14.13] |
|  |  |  |  |  |  |  |  |  |  |  |
| Former | Never + former | 5.43 | 5.17 | 5.16 | 3.84 | 3.54 | 3.48 | 4.17 | 3.84 | 3.79 |
|  |  | [2.41,12.22] | [2.32,11.51] | [2.31,11.54] | [2.83,5.19] | [2.62,4.80] | [2.57,4.71] | [3.13,5.54] | [2.89,5.11] | [2.85,5.04] |
|  |  |  |  |  |  |  |  |  |  |  |
| Former | Current | 2.60 | 1.81 | 1.70 | 5.76 | 4.29 | 4.03 | 5.48 | 3.97 | 3.75 |
|  |  | [0.53,12.75] | [0.37,8.95] | [0.34,8.42] | [3.41,9.72] | [2.51,7.31] | [2.33,6.96] | [3.33,9.03] | [2.40,6.59] | [2.24,6.28] |
|  |  |  |  |  |  |  |  |  |  |  |
|  |  |  |  |  |  |  |  |  |  |  |
| **Cerebrovascular diseases** | |  |  |  |  |  |  |  |  |  |
| Smoking | ST |  |  |  |  |  |  |  |  |  |
| Never | Never | Ref. | Ref. | Ref. | Ref. | Ref. | Ref. | Ref. | Ref. | Ref. |
|  |  |  |  |  |  |  |  |  |  |  |
|  |  |  |  |  |  |  |  |  |  |  |
| Never | Current | 2.27 | 1.84 | 1.75 | 1.25 | 1.04 | 1.00 | 1.49 | 1.21 | 1.15 |
|  |  | [0.84,6.15] | [0.68,4.98] | [0.65,4.71] | [0.62,2.51] | [0.51,2.13] | [0.49,2.05] | [0.84,2.62] | [0.67,2.17] | [0.64,2.06] |
|  |  |  |  |  |  |  |  |  |  |  |
| Never | Former | 2.21 | 2.13 | 2.06 | 1.09 | 1.01 | 0.99 | 1.32 | 1.23 | 1.19 |
|  |  | [0.67,7.26] | [0.64,7.08] | [0.63,6.79] | [0.47,2.55] | [0.43,2.39] | [0.42,2.34] | [0.66,2.62] | [0.61,2.46] | [0.59,2.39] |
|  |  |  |  |  |  |  |  |  |  |  |
| Current | Never + former | 2.30 | 1.77 | 1.57 | 1.55 | 1.40 | 1.34 | 1.80 | 1.55 | 1.46 |
|  |  | [1.53,3.45] | [1.16,2.70] | [1.02,2.41] | [1.16,2.07] | [1.03,1.90] | [0.99,1.82] | [1.44,2.25] | [1.22,1.96] | [1.14,1.86] |
|  |  |  |  |  |  |  |  |  |  |  |
| Current | Current | 1.62 | 1.13 | 0.99 | 1.72 | 1.45 | 1.29 | 1.63 | 1.32 | 1.17 |
|  |  | [0.34,7.78] | [0.23,5.51] | [0.21,4.67] | [0.49,6.08] | [0.40,5.21] | [0.35,4.77] | [0.61,4.36] | [0.48,3.57] | [0.43,3.18] |
|  |  |  |  |  |  |  |  |  |  |  |
| Former | Never + former | 1.19 | 1.19 | 1.11 | 1.14 | 1.12 | 1.11 | 1.16 | 1.14 | 1.11 |
|  |  | [0.77,1.85] | [0.77,1.84] | [0.72,1.72] | [0.91,1.42] | [0.89,1.41] | [0.88,1.39] | [0.95,1.41] | [0.93,1.39] | [0.91,1.36] |
|  |  |  |  |  |  |  |  |  |  |  |
| Former | Current | 2.32 | 2.01 | 1.73 | 1.63 | 1.42 | 1.38 | 1.78 | 1.52 | 1.44 |
|  |  | [1.02,5.25] | [0.84,4.79] | [0.73,4.11] | [0.95,2.79] | [0.82,2.46] | [0.80,2.40] | [1.13,2.79] | [0.95,2.43] | [0.90,2.29] |
|  |  |  |  |  |  |  |  |  |  |  |
| **Smoking related diseases ^g^** | |  |  |  |  |  |  |  |  |  |
| Smoking | ST |  |  |  |  |  |  |  |  |  |
| Never | Never | Ref. | Ref. | Ref. | Ref. | Ref. | Ref. | Ref. | Ref. | Ref. |
|  |  |  |  |  |  |  |  |  |  |  |
|  |  |  |  |  |  |  |  |  |  |  |
| Never | Current | 1.44 | 1.33 | 1.30 | 1.43 | 1.22 | 1.15 | 1.44 | 1.25 | 1.19 |
|  |  | [1.03,2.02] | [0.96,1.83] | [0.94,1.79] | [1.13,1.80] | [0.96,1.55] | [0.89,1.47] | [1.19,1.75] | [1.03,1.53] | [0.97,1.46] |
|  |  |  |  |  |  |  |  |  |  |  |
| Never | Former | 1.34 | 1.25 | 1.21 | 1.40 | 1.31 | 1.31 | 1.35 | 1.29 | 1.27 |
|  |  | [0.86,2.07] | [0.83,1.89] | [0.79,1.84] | [1.08,1.82] | [1.01,1.71] | [1.00,1.70] | [1.08,1.70] | [1.03,1.62] | [1.01,1.59] |
|  |  |  |  |  |  |  |  |  |  |  |
| Current | Never + former | 2.97 | 2.61 | 2.40 | 2.75 | 2.52 | 2.38 | 2.94 | 2.65 | 2.47 |
|  |  | [2.70,3.26] | [2.37,2.87] | [2.18,2.64] | [2.54,2.98] | [2.33,2.73] | [2.20,2.58] | [2.76,3.12] | [2.49,2.81] | [2.32,2.63] |
|  |  |  |  |  |  |  |  |  |  |  |
| Current | Current | 2.56 | 2.19 | 1.96 | 3.39 | 2.87 | 2.61 | 3.07 | 2.60 | 2.32 |
|  |  | [1.94,3.38] | [1.66,2.90] | [1.50,2.56] | [2.65,4.34] | [2.26,3.65] | [2.00,3.38] | [2.55,3.68] | [2.17,3.11] | [1.93,2.79] |
|  |  |  |  |  |  |  |  |  |  |  |
| Former | Never + former | 1.20 | 1.24 | 1.21 | 1.55 | 1.50 | 1.47 | 1.46 | 1.43 | 1.40 |
|  |  | [1.08,1.33] | [1.12,1.38] | [1.09,1.34] | [1.45,1.65] | [1.41,1.61] | [1.37,1.57] | [1.38,1.54] | [1.35,1.52] | [1.32,1.48] |
|  |  |  |  |  |  |  |  |  |  |  |
| Former | Current | 1.42 | 1.37 | 1.24 | 2.04 | 1.73 | 1.63 | 1.86 | 1.62 | 1.52 |
|  |  | [1.10,1.84] | [1.05,1.78] | [0.96,1.61] | [1.73,2.40] | [1.48,2.04] | [1.39,1.91] | [1.62,2.13] | [1.41,1.86] | [1.32,1.74] |
|  |  |  |  |  |  |  |  |  |  |  |
| **Other causes ^h^** | |  |  |  |  |  |  |  |  |  |
| Smoking | ST |  |  |  |  |  |  |  |  |  |
| Never | Never | Ref. | Ref. | Ref. | Ref. | Ref. | Ref. | Ref. | Ref. | Ref. |
|  |  |  |  |  |  |  |  |  |  |  |
|  |  |  |  |  |  |  |  |  |  |  |
| Never | Current | 1.83 | 1.72 | 1.68 | 1.37 | 1.33 | 1.27 | 1.51 | 1.45 | 1.39 |
|  |  | [1.22,2.72] | [1.15,2.58] | [1.11,2.54] | [1.05,1.79] | [1.01,1.75] | [0.97,1.65] | [1.21,1.90] | [1.15,1.83] | [1.10,1.74] |
|  |  |  |  |  |  |  |  |  |  |  |
| Never | Former | 1.34 | 1.28 | 1.20 | 0.94 | 0.95 | 0.94 | 1.07 | 1.06 | 1.04 |
|  |  | [0.73,2.45] | [0.71,2.31] | [0.69,2.09] | [0.66,1.33] | [0.66,1.35] | [0.66,1.35] | [0.78,1.46] | [0.78,1.45] | [0.76,1.42] |
|  |  |  |  |  |  |  |  |  |  |  |
| Current | Never + former | 2.06 | 1.77 | 1.60 | 1.66 | 1.62 | 1.55 | 1.81 | 1.68 | 1.57 |
|  |  | [1.82,2.33] | [1.55,2.01] | [1.40,1.82] | [1.48,1.86] | [1.45,1.82] | [1.38,1.74] | [1.67,1.97] | [1.55,1.82] | [1.45,1.71] |
|  |  |  |  |  |  |  |  |  |  |  |
| Current | Current | 2.32 | 1.95 | 1.68 | 1.74 | 1.77 | 1.65 | 2.01 | 1.89 | 1.70 |
|  |  | [1.58,3.43] | [1.32,2.88] | [1.11,2.55] | [1.07,2.82] | [1.09,2.90] | [0.99,2.75] | [1.49,2.72] | [1.40,2.56] | [1.24,2.32] |
|  |  |  |  |  |  |  |  |  |  |  |
| Former | Never + former | 1.09 | 1.18 | 1.14 | 1.14 | 1.14 | 1.12 | 1.12 | 1.14 | 1.12 |
|  |  | [0.95,1.24] | [1.03,1.36] | [1.00,1.32] | [1.05,1.24] | [1.05,1.23] | [1.03,1.21] | [1.04,1.20] | [1.06,1.22] | [1.04,1.20] |
|  |  |  |  |  |  |  |  |  |  |  |
| Former | Current | 1.49 | 1.54 | 1.37 | 1.13 | 1.05 | 1.02 | 1.21 | 1.13 | 1.08 |
|  |  | [1.01,2.20] | [1.04,2.29] | [0.92,2.04] | [0.90,1.42] | [0.83,1.33] | [0.80,1.29] | [0.99,1.47] | [0.92,1.39] | [0.88,1.33] |

^a^ [95% confidence interval]

^b^ All results are weighted. Participants with missing tobacco status, race/ethnicity, educational attainment, marital status or self-reported health status are excluded.

^c^ Hazard ratio adjusted for age

^d^ Hazard ratio adjusted for age, race/ethnicity, educational attainment, marital status, family income, region, and survey years

^e^ Hazard ratio adjusted for age, race/ethnicity, educational attainment, marital status, family income, region, survey years, BMI categories, and self-reported health status.

^f^ NE, not estimated; the number of deaths = 0.

^g^ Smoking related diseases: diseases of heart, malignant neoplasms, chronic lower respiratory diseases, cerebrovascular diseases, diabetes mellitus, and influenza and pneumonia.

^h^ Other causes: accidents, Alzheimer's disease, nephritis, nephrotic syndrome and nephrosis, and all other causes.

Supplemental Table 3. Hazard ratios ^a^ of all-cause mortality associated with tobacco users among men age 40-79 years in NHIS 1987, 1991, 1992, 1998, 2000 and 2005 ^b^

|  |  | Age 40-59 | | Age 60-79 | | Age 40-79 | |
| --- | --- | --- | --- | --- | --- | --- | --- |
|  |  | Excluding cigar/pipe status | Including cigar/pipe status | Excluding cigar/pipe status | Including cigar/pipe status | Excluding cigar/pipe status | Including cigar/pipe status |
| Smoking | ST ^c^ use |  |  |  |  |  |  |
|  |  |  |  |  |  |  |  |
| Never | Never | Ref. | Ref. | Ref. | Ref. | Ref. | Ref. |
|  |  |  |  |  |  |  |  |
|  |  |  |  |  |  |  |  |
| Never | Current | 1.41 | 1.38 | 1.26 | 1.25 | 1.29 | 1.28 |
|  |  | [1.06,1.88] | [1.03,1.84] | [1.04,1.54] | [1.03,1.53] | [1.09,1.53] | [1.08,1.51] |
|  |  |  |  |  |  |  |  |
| Never | Former | 1.12 | 1.11 | 1.14 | 1.13 | 1.14 | 1.12 |
|  |  | [0.80,1.57] | [0.79,1.56] | [0.91,1.42] | [0.90,1.41] | [0.94,1.38] | [0.93,1.36] |
|  |  |  |  |  |  |  |  |
| Current | Never + Former | 2.15 | 2.12 | 2.08 | 2.07 | 2.16 | 2.14 |
|  |  | [1.98,2.33] | [1.95,2.30] | [1.94,2.23] | [1.93,2.22] | [2.04,2.27] | [2.02,2.25] |
|  |  |  |  |  |  |  |  |
| Current | Current | 1.85 | 1.76 | 2.37 | 2.33 | 2.13 | 2.07 |
|  |  | [1.46,2.34] | [1.39,2.25] | [1.78,3.15] | [1.75,3.10] | [1.78,2.54] | [1.74,2.48] |
|  |  |  |  |  |  |  |  |
| Former | Never + Former | 1.19 | 1.18 | 1.33 | 1.32 | 1.29 | 1.28 |
|  |  | [1.10,1.30] | [1.08,1.29] | [1.26,1.40] | [1.25,1.40] | [1.24,1.36] | [1.22,1.35] |
|  |  |  |  |  |  |  |  |
| Former | Current | 1.34 | 1.31 | 1.43 | 1.41 | 1.39 | 1.37 |
|  |  | [1.06,1.68] | [1.04,1.65] | [1.24,1.64] | [1.22,1.63] | [1.24,1.57] | [1.21,1.55] |
|  |  |  |  |  |  |  |  |
| Cigar/pipe status |  |  |  |  |  |  |  |
|  |  |  |  |  |  |  |  |
| Never use |  |  | Ref. |  | Ref. |  | Ref. |
|  |  |  |  |  |  |  |  |
|  |  |  |  |  |  |  |  |
| Current use |  |  | 1.17 |  | 1.03 |  | 1.08 |
|  |  |  | [1.05,1.30] |  | [0.93,1.15] |  | [0.99,1.17] |
|  |  |  |  |  |  |  |  |
| Former use |  |  | 1.05 |  | 1.02 |  | 1.04 |
|  |  |  | [0.97,1.13] |  | [0.97,1.08] |  | [0.99,1.08] |
|  |  |  |  |  |  |  |  |
| Obs. |  | 22494 | 22494 | 13053 | 13053 | 35547 | 35547 |

^a^ [95% confidence interval]

^b^ All results are weighted. Participants with missing tobacco status, race/ethnicity, educational attainment, marital status or self-reported health status are excluded.

^c^ ST, smokeless tobacco
